# Alterations in Spectral Attributes of Surface Electromyograms after Utilization of a Foot Drop Stimulator during Post-Stroke Gait

**DOI:** 10.3389/fneur.2017.00449

**Published:** 2017-08-29

**Authors:** Rakesh Pilkar, Arvind Ramanujam, Karen J. Nolan

**Affiliations:** ^1^Human Performance and Engineering Research, Kessler Foundation, West Orange, NJ, United States; ^2^New Jersey Medical School, Newark, NJ, United States

**Keywords:** time–frequency analysis, functional electrical stimulation, wavelet transform, electromyography, spectral analysis

## Abstract

**Background:**

A foot drop stimulator (FDS) is a rehabilitation intervention that stimulates the common peroneal nerve to facilitate ankle dorsiflexion at the appropriate time during post-stroke hemiplegic gait. Time–frequency analysis (TFA) of non-stationary surface electromyograms (EMG) and spectral variables such as instantaneous mean frequency (IMNF) can provide valuable information on the long-term effects of FDS intervention in terms of changes in the motor unit (MU) recruitment during gait, secondary to improved dorsiflexion.

**Objective:**

The aim of this study was to apply a wavelet-based TFA approach to assess the changes in neuromuscular activation of the tibialis anterior (TA), soleus (SOL), and gastrocnemius (GA) muscles after utilization of an FDS during gait post-stroke.

**Methods:**

Surface EMG were collected bilaterally from the TA, SOL, and GA muscles from six participants (142.9 ± 103.3 months post-stroke) while walking without the FDS at baseline and 6 months post-FDS utilization. Continuous wavelet transform was performed to get the averaged time–frequency distribution of band pass filtered (20–300 Hz) EMGs during multiple walking trials. IMNFs were computed during normalized gait and were averaged during the stance and swing phases. Percent changes in the energies associated with each frequency band of 25 Hz between 25 and 300 Hz were computed and compared between visits.

**Results:**

Averaged time–frequency representations of the affected TA, SOL, and GA EMG show altered spectral attributes post-FDS utilization during normalized gait. The mean IMNF values for the affected TA were significantly lower than the unaffected TA at baseline (*p* = 0.026) and follow-up (*p* = 0.038) during normalized stance. The mean IMNF values significantly increased (*p* = 0.017) for the affected GA at follow-up during normalized swing. The frequency band of 250–275 Hz significantly increased in the energies post-FDS utilization for all muscles.

**Conclusion:**

The application of wavelet-based TFA of EMG and outcome measures (IMNF, energy) extracted from the time–frequency distributions suggest alterations in MU recruitment strategies after the use of FDS in individuals with chronic stroke. This further establishes the efficacy of FDS as a rehabilitation intervention that may promote motor recovery in addition to treating the secondary complications of foot drop due to post-stroke hemiplegia.

## Introduction

Stroke is one of the leading causes of serious and long-term disability, and foot drop is one of the most common disabling impairments resulting from hemiplegia due to stroke (1). Foot drop characterized by weakness and/or lack of voluntary control in the ankle and toe dorsiflexor muscles (2) can result in gait related deficiencies (decreased speed, a disruption in weight acceptance and transfer, asymmetry and instability), further limiting the activities of daily living (3, 4). The application of an ankle foot orthosis (AFO) to compensate for foot drop throughout the gait has been the common modality of treatment. Although AFOs have been shown to increase gait speed and functional ambulation (3, 5), as a rehabilitation intervention it is not targeted to restore muscle function (2).

Functional electrical stimulation (FES) has been evident as a targeted rehabilitation intervention that may promote motor recovery, especially when applied in a task-specific environment (6–9). FES applied to the common peroneal nerve through a foot drop stimulator (FDS) provides a focused excitation to the peroneal nerve to promote active ankle dorsiflexion during initial double support at heel strike, at pre-swing lift-off and during the swing phase of gait to sufficiently clear the foot (10–12). Using FDS to drive muscle groups in specific activation patterns during walking has been shown to improve strength, walking speed, spatiotemporal parameters, and retrain ankle dorsiflexor muscle [tibialis anterior (TA)] activation timings (2, 9, 13–16). These demonstrate the efficacy for FDS utilization in post-stroke rehabilitation, but they fail to precisely indicate how FDS technology can restore motor function (2, 12, 14, 16–20).

To understand the role of FDS-based gait rehabilitation in recovering motor function, it is important to understand the intrinsic electrophysiological modifications that may elicit the improvements in the muscle function. Surface electromyography is one of the most effective non-invasive tools, which provide easy access to the underlying physiological processes that cause the muscle to generate force, produce movement, and achieve any functional task (21). Electromyograms (EMG) data collected during gait can provide us with a quantitative measure of muscle activations and activation timings, which could be used to assess the level of improvement post-rehabilitation (10, 15, 22, 23). As a result, the application of various signal processing techniques to extract meaningful information from the EMG data has been an on-going process in stroke rehabilitation. To better understand the electrophysiological processes behind neuromuscular activations, it is essential to study these signals in time as well as frequency domain. Signal processing methods such as empirical mode decomposition (EMD) (24, 25) and wavelet analysis (26–28) have provided researchers tools to interpret non-stationary EMG data in time and frequency domain simultaneously. EMD has gained popularity for analyzing non-stationary signals and has been utilized in filtering EMG signals (25, 29, 30), time–frequency analysis (TFA) (25), fatigue analysis (31) due to its ability to decompose EMG signals into physically meaningful intrinsic mode functions (IMFs). Although EMD-based approach has advantages in analyzing EMG, time–frequency representations obtained using EMD-Hilbert transform could be excessively detailed, making it difficult to interpret, particularly for EMGs collected during dynamic movements such as gait. Although smoothing techniques have been suggested to obtain more continuous Hilbert spectrums, it has been suggested that such techniques may result in degradation of time–frequency resolution as well as physically meaningful content (24). In contrast, wavelet-based TFA provides more continues representation of the data and has been widely utilized to identify motor unit (MU) recruitment patterns (26), motor strategy patterns (28) and perform clinical assessments (27) during gait. In the current literature, these analyses have only been performed in individuals with cerebral palsy, diabetic neuropathy, ankle osteoarthritis, and healthy populations (26–28). The current investigation presents a novel application of wavelet-based TFA of EMG signals in individuals post-stroke during gait. In addition, wavelet-based TFA from lower extremity muscles is further analyzed to assess the neuromuscular changes occurring due to FDS-based gait retraining has not been done yet.

The purpose of this study was to apply a wavelet-based TFA approach to assess the neuromuscular changes in the TA, soleus (SOL), and gastrocnemius (GA) muscles after utilization of an FDS as a gait rehabilitation tool in individuals post-stroke. Changes in the spectral variable—instantaneous mean frequency (IMNF) and energies associated with bands of frequencies (25–300 Hz) extracted from time–frequency distributions of EMGs from TA, SOL, and GA muscles are compared to assess the alterations in MU recruitments post-FDS utilization. We hypothesize that the time–frequency distribution of targeted TA and indirectly stimulated SOL and GA muscles will show changes in time–frequency distribution (TFD) with increased mean IMNF and energy after utilization of an FDS.

## Materials and Methods

### Study Participants

Six individuals (age: 63.7 ± 10.6 years, height: 176.5 ± 6.2 cm, weight: 84.9 ± 8.3 kg, three right side affected, and three left side affected) with drop foot and hemiplegia secondary to stroke (142.9 ± 103.3 months post-stroke) were recruited for participation in this investigation. Hemiplegia was defined as paralysis affecting only one side of the body diagnosed by the participants’ treating physician. All participants (1) were at least 6 months post-stroke, prior to enrollment; (2) uninvolved lower limb had no history of injury or pathology; (3) were able to walk independently for 10 m without any assistive device; and (4) not currently participating in or is a minimum of 30 days post-inpatient or outpatient: stroke, cardiac, pulmonary, or any other physical rehabilitation on the lower extremities at time of enrollment. Individuals with severe cardiac disease, seizure disorders and/or orthopedic, neuromuscular, or neurological pathologies that would interfere with their ability to walk were excluded. All procedures performed in this investigation were approved by the Kessler Foundation Institutional Review Board, and written as well as informed consent was obtained from participants prior to study participation.

### Foot Drop Stimulator

**Figure 1:**
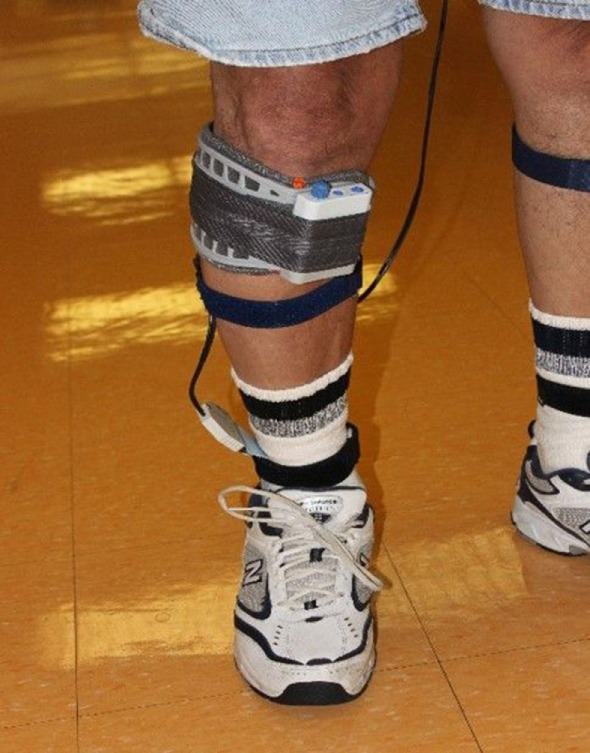
The Walkaide^®^ used for addressing foot drop.

All participants received a commercially available FDS (Walkaide^®^; Innovative Neurotronics, Inc., Austin, TX, USA) at their baseline visit for use during ambulation in the community as part of a larger multi-site clinical trial. The Walkaide^®^ is a battery operated single-channel, asymmetrical biphasic stimulator with programmable pulse width and frequency that was utilized during walking as an FES orthotic device (Figure 1). The technology is controlled by a tilt sensor and accelerometer to provide electrically induced muscle activation via two electrodes and electrode leads to dorsiflex the foot on the affected side at the appropriate time during gait. The small device [87.9 g, 8.2 cm (H) × 6.1 cm (W) × 2.1 cm (T)] was attached to a molded cuff located just below the knee, secured with a latch, and properly aligned using anatomical landmarks and visual indicators. The two electrodes were specifically placed near the head of the fibula, directly over the motor nerve and proximal musculature. Each participant used their own FDS device for daily ambulation in the community. Each device was custom programmed (stimulus intensity, timing and duration of muscle activation) during the gait cycle by a licensed clinician at baseline. The pulse width ranged from 25 to 300 μs and stimulation frequency ranged from 17 to 33 Hz.

### Testing Procedures

Participants completed 10 walking trials with (five trials) and without (five trials) the FDS at a self-selected pace on level ground (4.5 m) at baseline and following 6 months of FDS use for ambulation in the community. Participants wore shoes during all walking tests, and members of the study team provided non-contact guarding for safety. For consistency, the FDS was worn by the patient during all trials but switched off to prevent stimulation during the trials without the FDS. All walking trials without the FDS were used for subsequent analysis in the current investigation to measure the rehabilitative effect of the device.

### Data Acquisition and Processing

**Figure 2:**
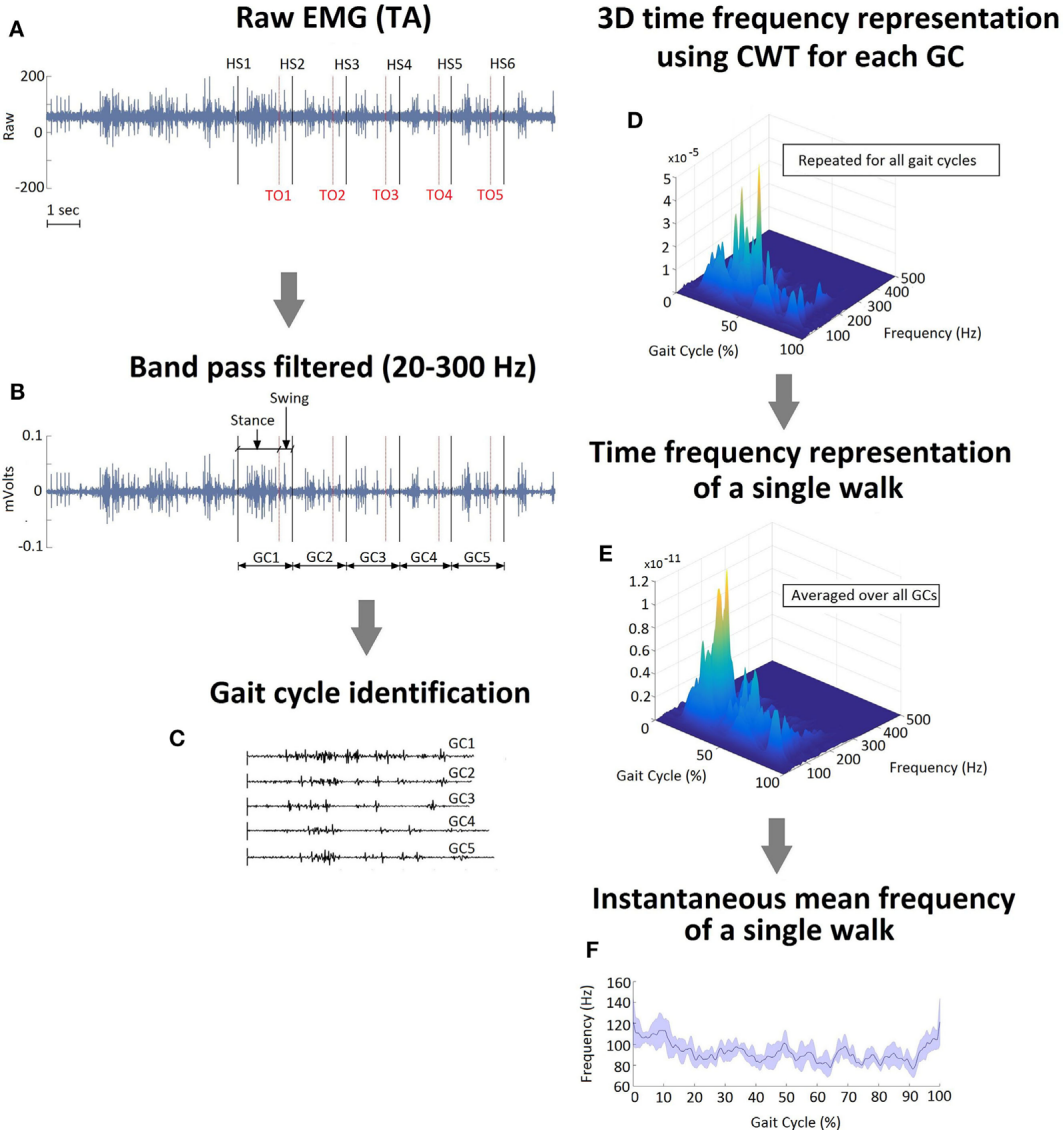
**(A)** Raw EMG signal recorded from tibialis anterior muscle on the affected side of a single representative participant during a single walk. Gait cycle (GC) events, heel strike and toe off, were identified. **(B)** Band pass filtered EMG with stance and swing events identified during a GC. **(C)** Raster plot representation of the individual GCs during a single walk. **(D)** A 3D time–frequency representation of GC1 data using continuous wavelet transform. **(E)** A time frequency representation of a single walk (averaged over five GCs). **(F)** Instantaneous mean frequency profile extracted from time–frequency representation shown in **(E)**. The shaded area in **(F)** represents the SD within the gait cycles during a single walk.

Wireless EMG data were collected bilaterally from the TA, SOL, and GA (medial head) muscles at 2,520 Hz using a Noraxon DTS system (Noraxon, Inc., Scottsdale, AZ, USA). All data were imported into Matlab (The Mathworks, Inc., Natick, MA, USA) for custom processing and analysis. EMG data during each walking trial (without FDS) were band pass filtered between 20 and 300 Hz and notch filtered at 60 Hz. The EMG amplitudes were normalized to the maximum voluntary contractions (MVCs). MVCs were collected for all selected muscles at the beginning of each testing session prior to walking trials and were done in accordance with SENIAM (32). Kinematic data were collected at 60 Hz (Motion Analysis Corporation, Santa Rose, CA, USA), and time synchronized with EMG data. During initial post-processing in Cortex software (Motion Analysis Corporation, Santa Rose, CA, USA) heel-strike and toe-off gait cycle events were identified for all trials based on the event of heel contact with the ground (or first foot contact in pathological gait) and the event of toe lift-off of the ground (or the last foot contact with the floor in pathological gait), respectively (Figure 2A). All EMG data were divided into gait cycles (GCs) for further analysis. A single GC consisted of consecutive heel-strike events of the same limb (Figures 2B,C). Each GC was subdivided into stance and swing based on heel-strike and toe-off events (Figure 2B). The total number of GC considered for analysis varied from a minimum of 18 to a maximum of 46 for baseline and a minimum of 11 to a maximum of 36 for follow-up visit across all participants.

### Data Analysis and Outcome Measures

#### Wavelet-Based TFA

One of the challenges of performing frequency or TFA of EMG data is the presence of non-stationarity (33). Non-stationarity in EMG data has been associated with several factors such as metabolic changes that induce fatigue, changes in muscle length as well as muscle force due to dynamic movements or the displacement of electrodes during movements (33). To better understand the underlying electrophysiological modifications that may cause improvements in the muscle function, we used a wavelet-based TFA approach, which has been shown to be suitable for analyzing non-stationary EMG data collected during dynamic muscle contractions (26–28).

The continuous wavelet transform (CWT) has the following general definition:

(1) $$W\left(a,b;x,\psi \right)=|a{|}^{-\frac{1}{2}}\overset{\infty }{\underset{-\infty }{\int }}x\left(t\right)\psi^{*}\left(\frac{t-b}{a}\right)dt$$

where ψ*(⋅) is a complex conjugate of the basic wavelet function, ψ, also called the mother wavelet. The parameter *a* is the dilation factor the controls the width of the wavelet, and *b* represents the translation of the origin thus controlling the location of the wavelet on time axis. The variable 1/a gives the frequency scale and *b* gives the temporal location of event for a signal *x*. In general, *W*(*a*,*b*;*x*,ψ) represents the energy of signal *x* of scale *a* at time, *t* = *b*. In this investigation, we performed the CWT using the analytic “bump” function as the mother wavelet of center frequency, *f*_c_ = 5/2π Hz using the command cwtft in Matlab. The scales, *a*, ranged from *f*_c_/(*f*_max_ × *dt*) to *f*_c_/(*f*_min_ × *dt*) where *f*_min_ and *f*_max_ were set to 1 and 500 Hz, respectively, as surface EMG signals are band-limited below 500 Hz. Also, *dt* represents the sampling duration.

For the current analysis, CWT was applied on the EMG data during each GC of varied time length to get the individual time–frequency representation, *W*. During the stance phase, swing phase and entire GC, *W* was individually resampled on the time axis to get the normalized representations (Figure 2D) for stance (0–100% of stance), swing (0–100% of swing), and entire GC (0–100% of entire GC). As a final step, TFDs were computed for both phases, during all walks (Figure 2E), and all participants were individually averaged to get a combined averaged TFD for each muscle.

##### Instantaneous Mean Frequency

Mean frequency (MNF) is an average frequency of a power density spectrum of a signal. IMNF can be computed from a TFD, *W*(*f, t*) as

(2) $$\text{IMNF}\left(t\right)=\frac{\sum_{j=1}^{M}f_{j}W\left(f_{j},t\right)}{\sum_{j=1}^{M}W\left(f_{j},t\right)}$$

Figure 2B shows the IMNF profile for a single normalized walk. IMNF was computed from the TFD of TA, SOL, and GA during stance and swing phases, and each were normalized to 0–100%. The averaged IMNF values were used for comparing the affected and unaffected side at each visit as well as comparing baseline and follow-up for each side.

##### TFD Energy (*E*)

The energy values were computed from the TFD, *W* obtained using CWT for a total of eleven frequency bands, each with a bandwidth of 25 Hz. The first band covered the frequencies from 25 to 50 Hz, the second band ranged from 51 to 75 Hz, and subsequent bands covering up to 300 Hz. Frequencies below 25 Hz and above 300 Hz were not considered for this analysis as the energies associated with these frequencies were negligible due to band pass filtering between 20 and 300 Hz. The TFD energy for each band was computed as a percentage of the total distribution energy as:

(3) $$E(\%)=100\times \frac{\sum_{f=f_{\text{a}}}^{f_{\text{b}}}W(f,t)}{\sum W(f,t)}$$

where *f*_a_ and *f*_b_ are frequency limits for the band. Energy values were computed separately for normalized stance and swing phases, for all the tested muscles at baseline and follow-up. The rationale behind dividing the entire time–frequency spectrum into several frequency bands was to isolate frequencies that showed significant changes in energy which may further help us understand the changes in MU recruitment strategies after the FDS utilization.

### Statistical Analysis

Paired sample *t*-tests were used to compare the differences in IMNF values of TA, SOL, and GA between the affected and unaffected sides. To evaluate the effect of FDS utilization on TA, SOL, and GA, IMNF and energy (*E*) values at baseline and follow-up visits were also compared using paired sample *t*-tests. Significance level was set to *p* < 0.05 for all statistical analyses.

## Results

**Figure 3:**
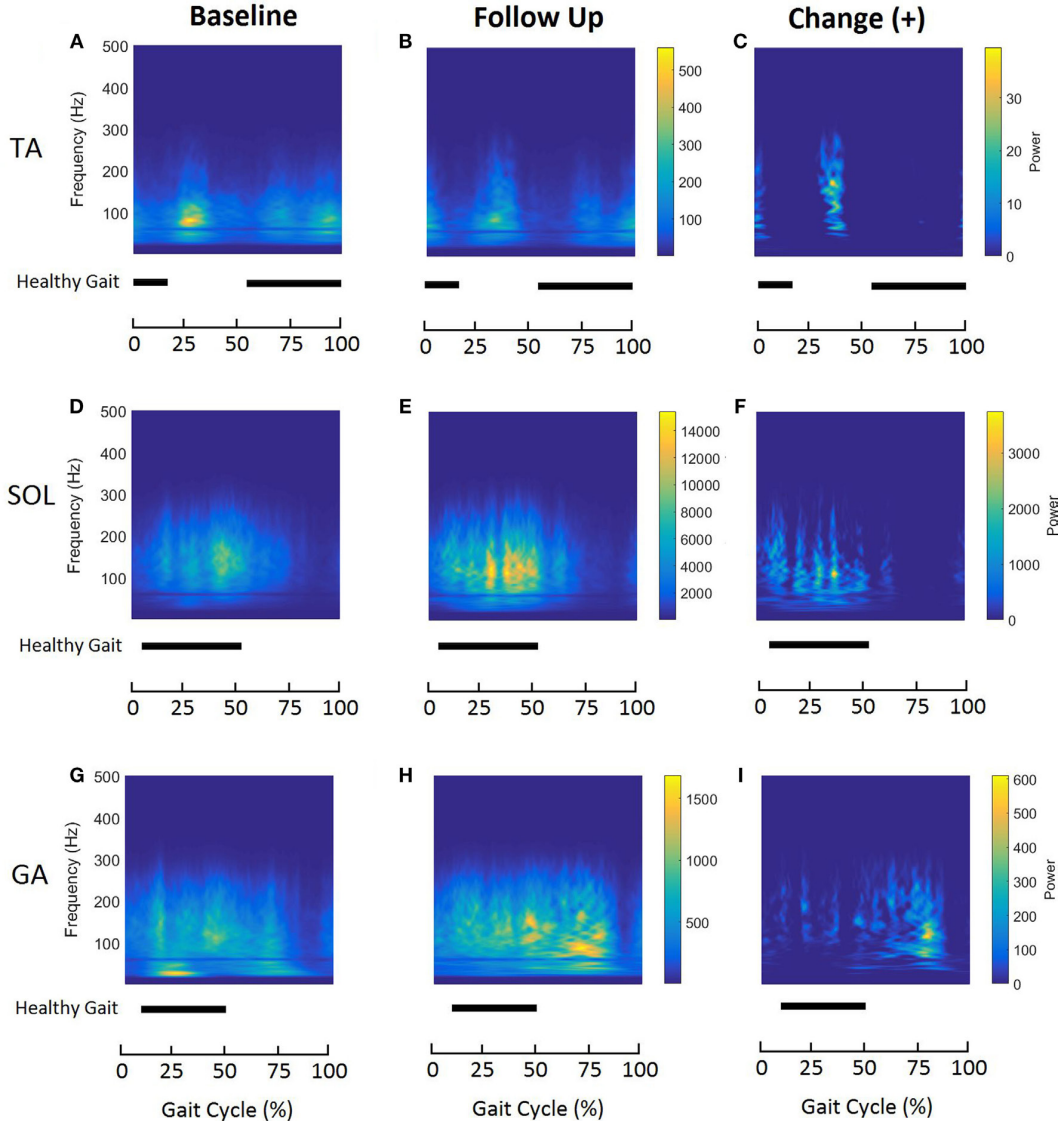
Averaged time–frequency representations of affected tibialis anterior **(A–C)**, soleus **(D–F)**, and gastrocnemius **(G–I)** muscles during normalized gait at baseline (left column) and follow-up (middle column) for all participants (*n* = 6). The right column represents the positive changes in the distributions after follow-up. Energy levels are presented as a color bar for each muscle. Horizontal bars indicate activation (ON–OFF) for the same muscle during normalized healthy gait, reported by Perry and Burnfield (22).

Time–frequency plots show distinct patterns of muscle activation and energy association with different phases of the GC for all tested muscles on the affected side (Figure 3). During healthy gait, the TA muscle produces two bursts of activation during the normalized gait cycle with initial activation occurring between 0 and 12% GC (during initial double support) and the second burst occurring at 55 and 100% of GC (during swing) (22). Figures 3A,B show similar patterns of TA activation at baseline and follow-up; however, the initial burst of activation appears to be prolonged for stroke participants compared to what is observed in healthy gait. At baseline, the highest energy is localized between 20 and 40% of GC and is associated with frequencies between 75 and 100 Hz. Furthermore, the TA inhibitory (inactive) period, which is usually apparent in healthy gait during 13–54% of GC, seems to be indistinct at baseline for stroke participants. At follow-up, time–frequency plot show alterations in TA activation (Figures 3B,C). This is characterized by (1) an increase in the energies during 0–10% and 30–40% of GC (Figure 3C) and (2) the presence of clear inhibition during approximately 50–70% of GC shown by negligible energy content (Figure 3B).

Soleus is active during 6–52% of GC during healthy gait (22). In the case of stroke gait, the energy contained within the SOL muscle is approximately between 5 and 75% of GC (Figures 3D,E). At follow-up, TFD of the SOL shows increased energies associated with frequencies ranging from 75 to 200 Hz (Figure 3F).

Time–frequency plots of the affected GA show a clear shift in the peak energy localization during normalized gait (Figures 3G,H). At baseline, the energies are localized at low frequencies (<50 Hz) between 20% and 30% of GC, suggesting the predominant use of slow-twitch MU (Figure 3G). However, after 6 months of FDS utilization, the energy content shows a clear shift toward higher frequencies (50–150 Hz) (Figures 3H,I). As seen with TA and SOL, GA time–frequency plots for stroke participants show wide energy spread compared to healthy gait.

**Figure 4:**
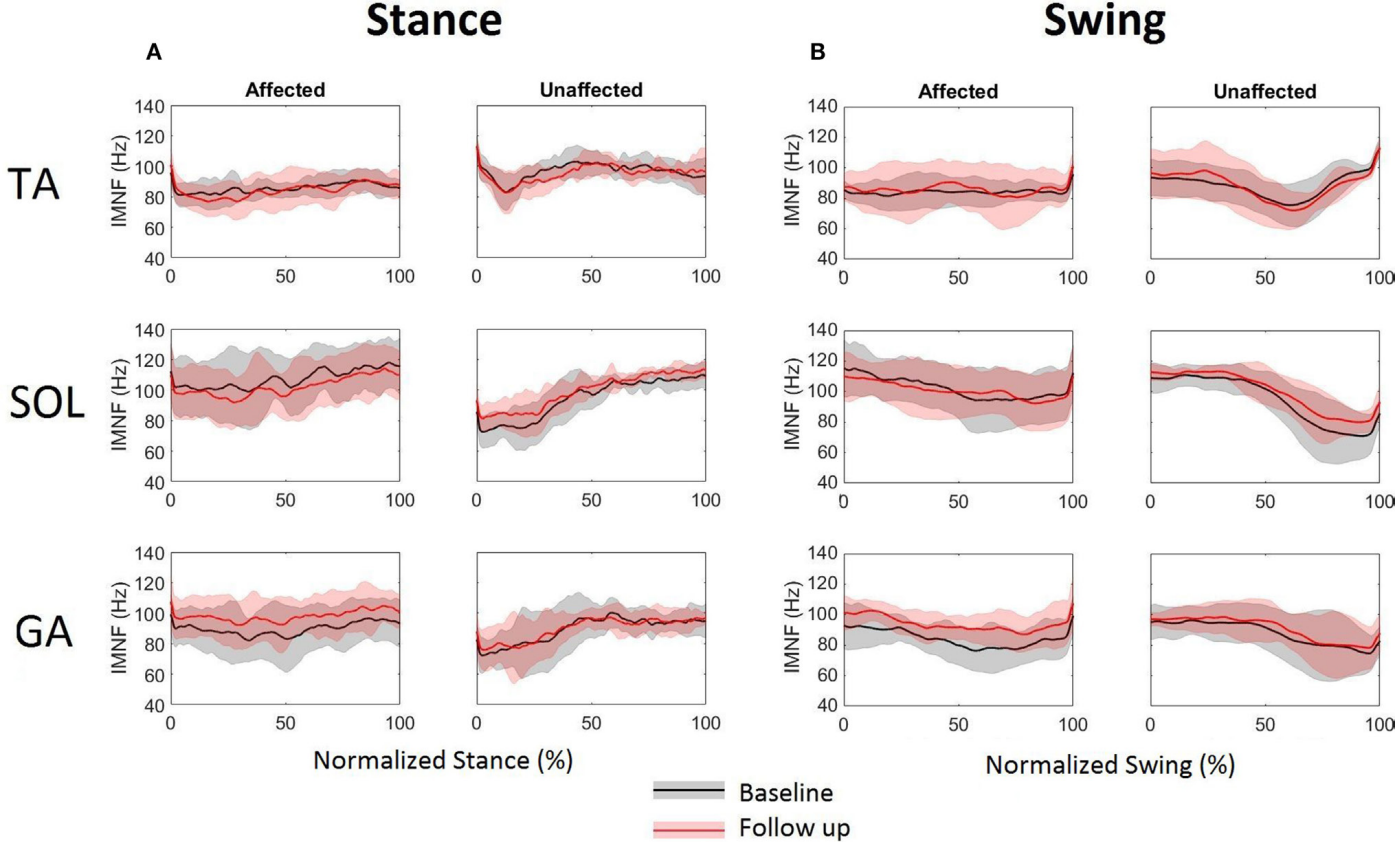
Instantaneous mean frequency profiles extracted from time–frequency distributions of tibialis anterior, soleus, and gastrocnemius EMG for affected (left column) and unaffected (right column) sides during normalized **(A)** stance and **(B)** swing at baseline and follow-up. The solid lines represent the mean and the shaded areas represent the ±SD across six stroke participants.

To quantify the time–frequency distributions, IMNF was computed during the normalized (0–100%) stance and swing using Eq. 2. Figure 4 shows difference in the IMNF trajectories between the affected and unaffected side, for all three muscles during baseline and follow-up visits. The unaffected TA has an initial decrease in the averaged IMNF profile approximately during 0–13% of normalized stance followed by a gradual increment and a plateau. During normalized swing, the unaffected TA shows a gradual decrease up to 63% followed by an increase in the averaged IMNF profile. Such a pattern is not apparent for the affected TA during normalized stance as well as swing. The averaged IMNF profiles of the unaffected SOL and GA muscles, being less impaired, show increasing trend in MNF with time during normalized stance (Figure 4A, right column) and show decreasing trend with time during normalized swing (Figure 4B, right column). Such a characteristic response is not seen on the affected side (Figures 4A,B, left columns, respectively). Moreover, it is observed that there is higher variability in IMNF profiles on the affected side across all participants compared to the unaffected side, particularly during normalized stance.

Table 1 presents the mean IMNF values computed during normalized stance and swing for each tested muscle bilaterally during both testing sessions. The mean IMNF values computed from the TA during normalized stance on the affected side were significantly lower than the unaffected side at baseline (*p* = 0.026) as well as follow-up (*p* = 0.038) (Table 1). There were no significant differences in mean IMNF for SOL and GA between affected and unaffected sides during stance (refer to Table 1—*P*_a_ values). The affected GA muscle showed a significant increment in the mean IMNF values during swing at follow-up (*p* = 0.017). There were no significant differences for the TA and SOL on each side during swing between baseline and follow-up (refer to Table 1—*P*_b_ values).

**Table 1:** Comparison of mean IMNF of TA, SOL, and GA muscles during normalized stance and swing phases of gait cycle for individuals with stroke (*n* = 6).

		Stance			Swing		
		Baseline	Follow-up	*P*_b_ value	Baseline	Follow-up	*P*_b_ value
TA	Affected	83. 8 (6.8)	82.6 (9.3)	0.715	82.9 (7.0)	84.5 (14.1)	0.842
	Unaffected	98.6 (6.7)	95.3 (2.8)	0.518	88.9 (8.1)	87.7 (8.4)	0.926
	***P*_a_ value**	0.026*	0.038*		**0.366**	**0.796**	
SOL	Affected	111.2 (19.2)	102.5 (14.1)	0.451	106.2 (17.6)	100.2 (13.2)	0.867
	Unaffected	95.3 (4.7)	96.7 (8.1)	0.118	96.9 (8.5)	99.7 (3.8)	0.072
	***P*_a_ value**	**0.201**	**0.713**		**0.366**	**0.847**	
GA	Affected	91.1 (11.5)	97.2 (11.7)	0.116	85.6 (8.6)	92.7 (7.9)	0.017*
	Unaffected	94.6 (18)	88.1 (9.4)	0.944	94.2 (19.8)	90.4 (8.9)	0.646
	***P*_a_ value**	**0.943**	**0.084**		**0.606**	**0.476**	

*P_a_: p- values computed using paired t-test for comparing affected and unaffected side for each visit*.

*P_b_: p- values computed using paired t-test for comparing baseline and follow-up visits for each side*.

*p < 0.05.

**Figure 5:**
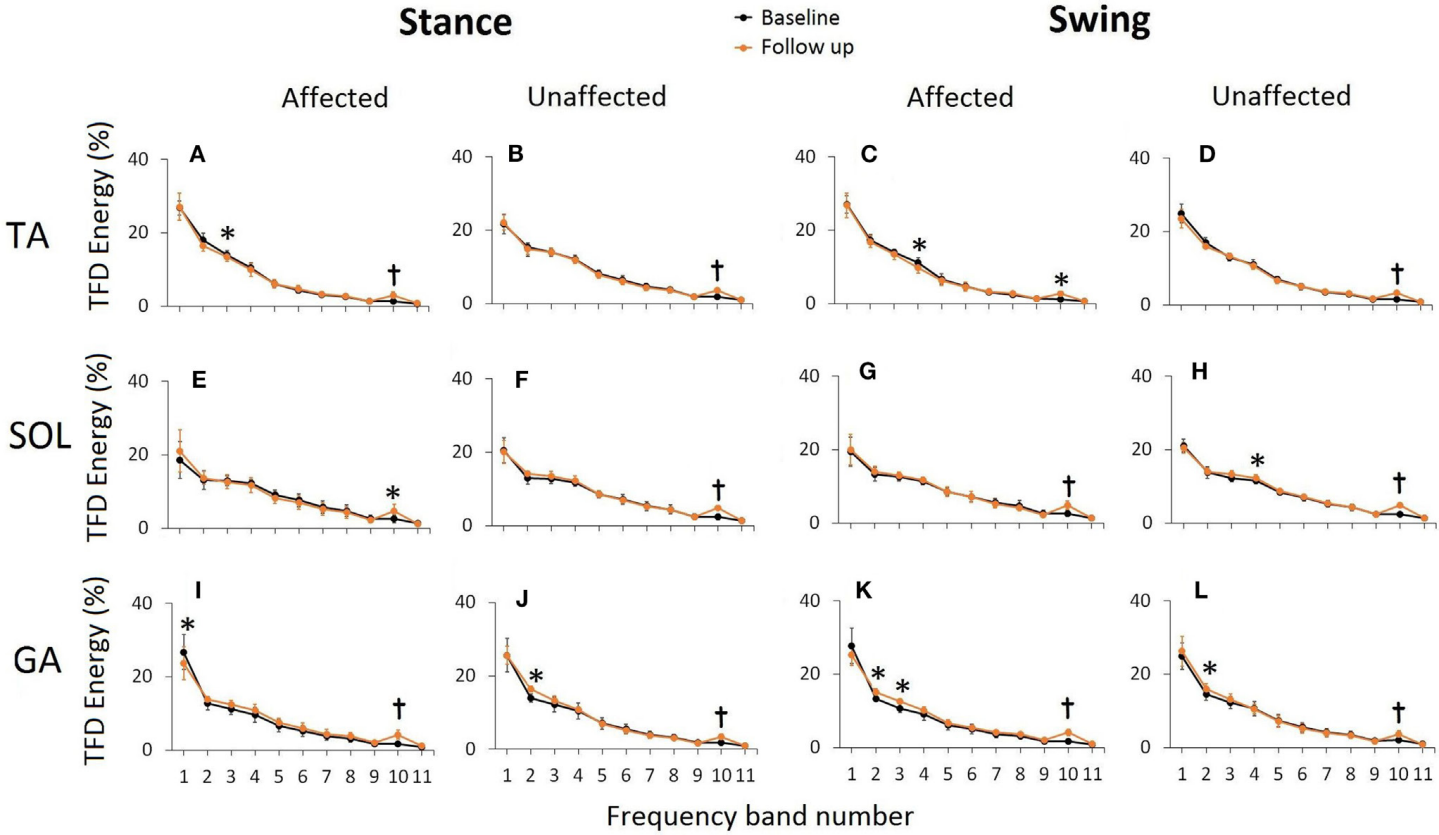
Time frequency distribution (TFD) energies associated with 11 predefined frequency bands during normalized stance and swing at baseline and follow-up for TA **(A–D)**, SOL **(E–H)**, and GA **(I–L)**. TFD energy in each band is presented as a percentage of total TFD energy during a normalized gait averaged across six stroke participants. Error bars represent ±SD. Significant differences are shown as *(*p* < 0.05) and t(*p* < 0.005). The frequencies associated with the predefined bands are 1: 25–50 Hz, 2: 51–75 Hz, 3: 76–100 Hz, 4: 101–125 Hz, 5: 126–150 Hz, 6: 151–175 Hz, 7: 176–200 Hz, 8: 201–225 Hz, 9: 226–250 Hz, 10: 251–275 Hz, and 11: 276–300 Hz.

To further quantify the time–frequency distribution plots and evaluate the effect of FDS utilization on muscle activation patterns, energies contained within eleven bands, each of 25 Hz bandwidth were computed in terms of percent of total energy and compared between the two visits. In general, the highest percent of energy was contained in low frequency bands and energies decreased as the frequency in the signal increased (Figure 5). Of all the energy bands, band 10 (250–275 Hz) was the most consistent in showing significant changes in energies for all three muscles bilaterally during normalized stance and swing, after FDS utilization. On the affected side, the TA showed a significant decrease in energies in band 3 (75–100 Hz) during stance (*p* = 0.04) and band 4 (101–125 Hz) during swing (*p* = 0.038) at follow-up. The unaffected SOL showed a significant increase (*p* = 0.025) in energy in band 4 (101–125 Hz) during swing. The affected GA showed a significant decrease in band 1 (25–50 Hz) during stance and showed a significant increase in energies associated with bands 2 and 3 (51–100 Hz) (*p* = 0.007 and *p* = 0.034, respectively) during swing. On the unaffected side, GA showed significant energy increments in band 2 (50–75 Hz) during stance (*p* = 0.008) as well as swing (*p* = 0.03).

## Discussion

Previous assessments to evaluate the efficacy of an FDS in gait retraining have focused on walking speed, spatiotemporal changes and muscle activation timings (2, 11, 12, 15, 16). The purpose of this study was to apply a wavelet-based TFA approach to assess the alterations in neuromuscular activations of TA, SOL, and GA muscles after utilization of an FDS during gait in individuals with stroke. FDS stimulates the common peroneal nerve which innervates the TA muscle. However, electrical stimulations are not spatially restricted to only stimulated nerves or muscles but also travel and stimulate neighboring muscles due to the electrical conductivity, interconnection within peripheral nervous system and physiological composition of muscles. Hence, although the TA received a targeted stimulation, GA and SOL are also indirectly stimulated during gait. In this study, the walking trials without FDS were used for analysis purposes to potentially examine the training or therapeutic effect of FDS utilization over the 6-month intervention period. Our results begin to demonstrate that utilization of FDS provides FES-based training not only to the TA but also to the GA and SOL muscles. As a result, the changes seen in time–frequency representations, IMNF and energies could be the training effect characterized by alterations in muscle activation patterns.

### Application of CWT

Time–frequency representations of TA, SOL, and GA muscles of the affected side showed altered neuromuscular activation during normalized gait post-FDS utilization. These alterations were indicated by increase in the signal energy (for TA and SOL), shifting of localized energy content on the time and frequency axis (for GA) and presence of inhibition (for TA) during normalized gait. The TA on both sides showed energy localization below 100 Hz on the frequency axis. This could suggest that predominantly slow MUs of the TA were recruited during gait due to the neuromuscular impairment as a result of foot drop. A shift in the peak energy localization on the frequency axis (from <50 to >100 Hz) for GA muscle on the affected side could be due to the change in MU recruitment strategies (slow to fast) post-FDS usage. However, there are potentially several reasons that may cause the above changes. The increase in the energy content could be directly related to an increase in the neuromuscular activation, which could be the result of FDS-based retraining. Motor learning using FES is thought to work through the creation of positive feedback of residual myoelectric activity acting to increase long-term potentiation of the corticomuscular connection (6–8, 16). Active repetitive movement training in combination with task-specific FES (such as gait) has shown to be perhaps the most promising use of FES for the facilitation of motor recovery (8). Specifically cyclic or EMG-based FES activation that acts to promote the movement goal in combination with voluntary effort has been found to produce greater physiological and functional gains than FES alone (9). In this study, the participant used the FDS in combination with volitional efforts to perform a cyclic activity—gait that may have promoted the changes in the time–frequency domain for TA, SOL, and GA muscles.

### Changes in IMNF

The spectral parameters extracted using wavelet-based analysis have shown to be associated with fatigue, muscle properties (fiber types), and MU recruitment strategies (28, 34, 35). The spectral measures such as IMNF and instantaneous median frequencies (IMDFs) have been extensively used is assessing muscle fatigue during isometric muscle contractions, as IMNF/IMDFs show a clear downward shift in frequency with respect to time as a result of fatigue (36). Furthermore, muscle fatigue may also cause decrease in signal power at high frequencies, a small increase in signal power at low frequency due to alterations in recruitment firing and conduction velocities, and synchronization of the signals (37). During normalized stance as well as swing, TA, SOL, and GA muscles of unaffected side showed distinct patterns of IMNF compared to affected side as shown in Figure 4. Mean IMNF values for the TA EMG during normalized stance was significantly lower for the affected side than unaffected side during baseline as well as follow-up visits. With the TA muscle being impaired due to foot drop post-stroke, the decrease in the mean IMNF could be due to impairment in recruitment of fast MU during stance. During swing, this difference was insignificant.

Affected GA muscle showed a significant increase in the mean IMNF values during swing at follow-up. This increment in mean IMNF can be correlated to the shift in peak energy localization to higher frequencies (Figures 3H,I) in TFD for affected GA muscle at follow-up. This could be due to either increment in recruitments of number of MUs within band 2 (51–75 Hz), band 3 (76–100 Hz), and band 10 (251–275 Hz), or increment in outputs generated by MUs of firing rates lying among bands 2, 3, and 10 or all. Although our analysis does not identify the exact MUs that underwent such alterations, it definitely identifies the frequency bands within which the alterations in recruitment strategies occurred as a result of FDS-based training in cyclic, task-specific environment such as gait.

The difference between the affected and unaffected sides was demonstrated by IMNF profiles for SOL and GA (Figure 4). The IMNF profiles were influenced by the dynamic movement of ankle and knee during gait. During stance, SOL and GA act as plantar flexors to “lock” the ankle so the limb and foot rotate on the forefoot rocker (22). As a result, the increase in IMNF from 0 to 60% during normalized stance for both GA and SOL on the unaffected side could not be associated with the ankle joint positional changes but may suggest the recruitment of MU in the order of slow to fast MUs. Conversely, decrease in IMNF between approximately 50 and 80% of normalized swing on the unaffected side could be related to start of the inhibition/de-recruitment of MU from the order of fast to slow MUs. It has been shown that, at the upper limit of MU recruitment, the mean or median frequencies should reach a plateau (34). This confirms with our IMNF profiles for SOL and GA on the unaffected side during normalized gait where IMNF gradually increases on EMG onset, plateaus during peak activation and subsides prior to the end of the activations.

### Changes in the TFD Energies

Previously, foot orthoses have been shown to alter the energies associated with high frequency bands of lower extremity muscle activity in recreational runners (38). We found that the energies associated with the second highest frequency band of 250–270 Hz significantly increased for all tested muscles during normalized gait. There are several physiological factors such as conduction velocity of fibers within MU, shape of intracellular action potentials, number of MU recruited, MU discharge rates, and MU synchronization that may affect the generation of an EMG signal (34). Furthermore, the amplitude of the surface EMG is related to the net MU activity: the recruitment and the discharge rates of active MUs (34). As a result, the energy changes associated with a frequency band in a TFD could be related to either increase or decrease in the amplitudes of MUs with firing rates within that band or the number of MUs recruited, e.g., GA muscle on the affected side showed a significant decrease in the energies associated with low frequencies (25–50 Hz) and significant increase in energies between 50 and 100 Hz. These alterations in the MU recruitment patterns (slower to faster MUs) could be a result of post-stroke spontaneous recovery or long-term recovery due to neuroplasticity or a rehabilitation intervention that promotes motor learning and recovery. In the current investigation, the participants represent a chronic stroke population with duration since last stroke ranging from 33 to 244 months. Hence, the possibility of such alterations in MU recruitment due to spontaneous recovery is unlikely. Therefore, significant energy changes specifically between 250 and 270 Hz could suggest the effect of FDS-based intervention. It should be noted that the purpose of this investigation was not to uniquely identify the activity of individual MUs using the sEMG but to detect the alterations in spectral attributes of EMG which may suggest the changes in recruitment strategies resulting from FDS-based gait retraining.

The SD of IMNF profiles (Figure 4) and the TFD energy values (Figure 5) at the lower frequency bands (bands 1–4) showed higher variability across the participants. The variability in the EMG data and its spectral correlates across participants can be associated with variability in several factors such as affected areas of the brain, months post-stroke, and compensatory gait strategies. Another factor that may have contributed to variability in the data is FDS parameters (stimulation intensity, pulse width, stimulation frequency, and frequency of FDS use). FDS stimulation frequency ranged from 19 to 33 Hz, and pulse width ranged from 25 to 300 µs. Selection of these parameters was done by a trained clinician based on the gait cycle timing, amount of dorsiflexion needed to lift the foot, gait speed, and patient discomfort threshold. Variability in FDS parameters, FDS dosing, as well as a small sample size may have led to the lack of significant differences for mean IMNF values of affected TA and SOL.

### Limitations and Future Work

The changes in EMG and its correlates bilaterally could be further explained by potentially altered spatiotemporal and kinematic parameters after FDS utilization. However, these variables were not analyzed in the current investigation. Future research will explore the relationship between CWT-based time–frequency measures, spatiotemporal parameters, and kinematic variables. The current investigation is also limited by a smaller sample size. Future research will focus on assessing training effects of FDS-based gait intervention on a larger sample and will also include a control group to isolate the effect of FDS intervention on the spectral properties of EMG data.

## Conclusion

This investigation demonstrates the applicability of wavelet-based TFA of EMG data to assess the neuromuscular alterations after the use of FDS during gait for individuals with chronic stroke. The changes in time–frequency distributions, IMNF, and energies may suggest the alterations in MU recruitment after FDS utilization during gait, thus further establishing the utility of FDS as a rehabilitative intervention that may promote motor recovery secondary to treating foot drop resulting from hemiplegia post-stroke.

## Ethics Statement

This study was carried out in accordance with the recommendations of Kessler Foundation Institution Review Board (IRB) with written informed consent from all subjects. All subjects gave written informed consent in accordance with the Declaration of Helsinki. The protocol was approved by the Kessler Foundation’s IRB Committee.

## Author Contributions

RP assisted in study design, data collection, performed EMG analysis, and prepared the manuscript. AR assisted with data collection, processed the motion analysis data and assisted with manuscript preparation. KN assisted in study design, data collection, data interpretation, and manuscript preparation.

## Conflict of Interest Statement

The authors declare that the research was conducted in the absence of any commercial or financial relationships that could be construed as a potential conflict of interest. The reviewer, CD, and handling editor declared their shared affiliation.
